# Complete Heart Block Unmasking Transthyretin Cardiac Amyloidosis: A Multimodality Imaging Case Report

**DOI:** 10.7759/cureus.112834

**Published:** 2026-07-17

**Authors:** Hafsa Erregui, Mehdi Moujahid, Nadia Loudiyi, Mohamed Malki, Zouhair Lakhal, Aatif Benyass

**Affiliations:** 1 Cardiology, Mohammed V Military Teaching Hospital, Rabat, MAR; 2 Cardiology, Ibn Sina University Hospital Center, Rabat, MAR

**Keywords:** cardiac amyloidosis, complete heart block, multimodality imaging, syncope, transthyretin cardiac amyloidosis

## Abstract

Transthyretin cardiac amyloidosis (ATTR-CM) is an increasingly recognized infiltrative cardiomyopathy characterized by progressive myocardial amyloid deposition, leading to heart failure, arrhythmias, and conduction abnormalities. Recent studies suggest that ATTR-CM is not uncommon among older patients hospitalized with heart failure with preserved ejection fraction and left ventricular hypertrophy, highlighting that the disease remains substantially underdiagnosed. Conduction disturbances are common during the course of the disease, and advanced atrioventricular conduction disease may require permanent pacemaker implantation; however, complete heart block as the initial presentation remains uncommon. We report the case of a 70-year-old woman who presented with exertional syncope and was found to have complete atrioventricular block with a wide-QRS escape rhythm, requiring emergency temporary pacing followed by permanent dual-chamber pacemaker implantation. Transthoracic echocardiography demonstrated marked biventricular hypertrophy, biatrial enlargement, restrictive left ventricular filling pattern, and severely impaired global longitudinal strain with a characteristic apical sparing pattern, raising a strong suspicion of cardiac amyloidosis. Cardiac MRI revealed diffuse biventricular subendocardial late gadolinium enhancement and abnormal myocardial nulling, highly suggestive of amyloid infiltration. Hematological investigations excluded light-chain amyloidosis, and technetium-99m hydroxymethylene diphosphonate scintigraphy demonstrated Perugini grade 3 myocardial uptake, establishing the non-biopsy diagnosis of ATTR-CM. Tafamidis therapy was subsequently initiated. This case highlights complete heart block as an uncommon initial presentation of ATTR-CM. It underscores the pivotal role of multimodality imaging in achieving an early and accurate diagnosis, thereby enabling timely initiation of disease-modifying therapy.

## Introduction

Cardiac amyloidosis (CA) is an infiltrative cardiomyopathy characterized by the extracellular deposition of misfolded amyloid fibrils within the myocardium, resulting in progressive ventricular thickening, heart failure, arrhythmias, conduction disturbances, and restrictive ventricular filling physiology [1]. Among its different subtypes, transthyretin CA (ATTR-CM) is now recognized as an important cause of heart failure in older adults owing to advances in multimodality imaging and heightened clinical awareness [2]. A prospective screening study demonstrated that wild-type ATTR-CM was identified in 13.3% of patients aged ≥60 years hospitalized with heart failure with preserved ejection fraction and left ventricular hypertrophy, highlighting that the disease remains substantially underdiagnosed in older adults [3]. In addition to cardiac involvement, extracardiac manifestations, including bilateral carpal tunnel syndrome, lumbar spinal stenosis, spontaneous distal biceps tendon rupture, peripheral or autonomic neuropathy, orthostatic hypotension, and gastrointestinal dysmotility, often precede cardiac manifestations by several years and represent important red flags that should prompt consideration of ATTR-CM, particularly by non-cardiology specialists [2,4].

Conduction abnormalities are common in ATTR-CM and result from progressive amyloid infiltration of the sinoatrial node, atrioventricular node, and His-Purkinje system [4]. First-degree atrioventricular block, bundle branch block, atrial arrhythmias, and other conduction disturbances are frequently encountered, and advanced conduction disease may ultimately require implantation of a permanent pacemaker [5]. However, complete heart block as the initial clinical presentation of ATTR-CM remains uncommon and has been reported mainly in isolated case reports and small case series [4,5].

We report the case of a 70-year-old woman who presented with syncope secondary to complete atrioventricular block, which ultimately led to the diagnosis of ATTR-CM through multimodality imaging and noninvasive diagnostic confirmation. This case highlights the importance of considering CA in elderly patients presenting with unexplained advanced conduction disease. It also underscores the pivotal role of multimodality imaging in enabling rapid and accurate diagnosis, thereby enabling timely initiation of disease-modifying therapy.

## Case presentation

A 70-year-old woman with no prior history of hypertension, diabetes mellitus, dyslipidemia, smoking, valvular heart disease, cardiomyopathy, chronic kidney disease, or conduction abnormalities presented to the emergency department after her first episode of exertional syncope. She was not taking any medications known to impair atrioventricular conduction. A detailed clinical history revealed no extracardiac manifestations or other clinical red flags suggestive of transthyretin amyloidosis, including bilateral carpal tunnel syndrome, lumbar spinal stenosis, spontaneous distal biceps tendon rupture, peripheral or autonomic neuropathy, orthostatic hypotension, gastrointestinal dysmotility, unexplained weight loss, or a family history of amyloidosis or unexplained cardiomyopathy.

The syncopal episode occurred abruptly during physical exertion while walking, without prodromal symptoms such as dizziness, nausea, diaphoresis, or palpitations. The loss of consciousness was transient, with spontaneous and complete recovery and no postictal confusion, tongue biting, or urinary incontinence. She denied chest pain, dyspnea, orthopnea, or lower-extremity edema. She had no history of fever, recent infection, or constitutional symptoms.

On admission, the patient was hemodynamically stable except for marked bradycardia. Her blood pressure was 170/80 mmHg, heart rate was 28 beats per minute, respiratory rate was 16 breaths per minute, and oxygen saturation was 96% on room air.

Physical examination revealed normal heart sounds without any audible murmurs, particularly none suggestive of aortic stenosis. Pulmonary auscultation was unremarkable, with no crackles or signs of congestion. There were no signs of heart failure, and the remainder of the physical and neurological examinations was unremarkable.

**Figure 1:**
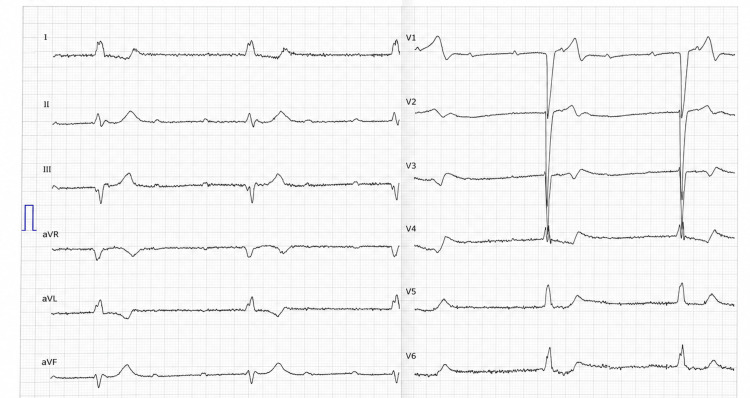
Admission electrocardiogram showing complete atrioventricular block A 12-lead electrocardiogram obtained on admission demonstrates complete atrioventricular block with atrioventricular dissociation and a slow, wide-QRS ventricular escape rhythm at 28 beats/min (QRS duration: 209 ms), consistent with an infra-Hisian escape rhythm. Interpretation of ischemic ST-segment and T-wave abnormalities was limited because of the wide-QRS ventricular escape rhythm.

A 12-lead electrocardiogram demonstrated complete atrioventricular block with atrioventricular dissociation and a wide-QRS ventricular escape rhythm at 28 beats/min (QRS duration: 209 ms), consistent with an infra-Hisian escape rhythm (Figure 1). Because of the wide-QRS ventricular escape rhythm, ischemic ST-segment and T-wave abnormalities could not be reliably assessed. Syncope secondary to complete heart block was therefore diagnosed.

**Figure 2:**
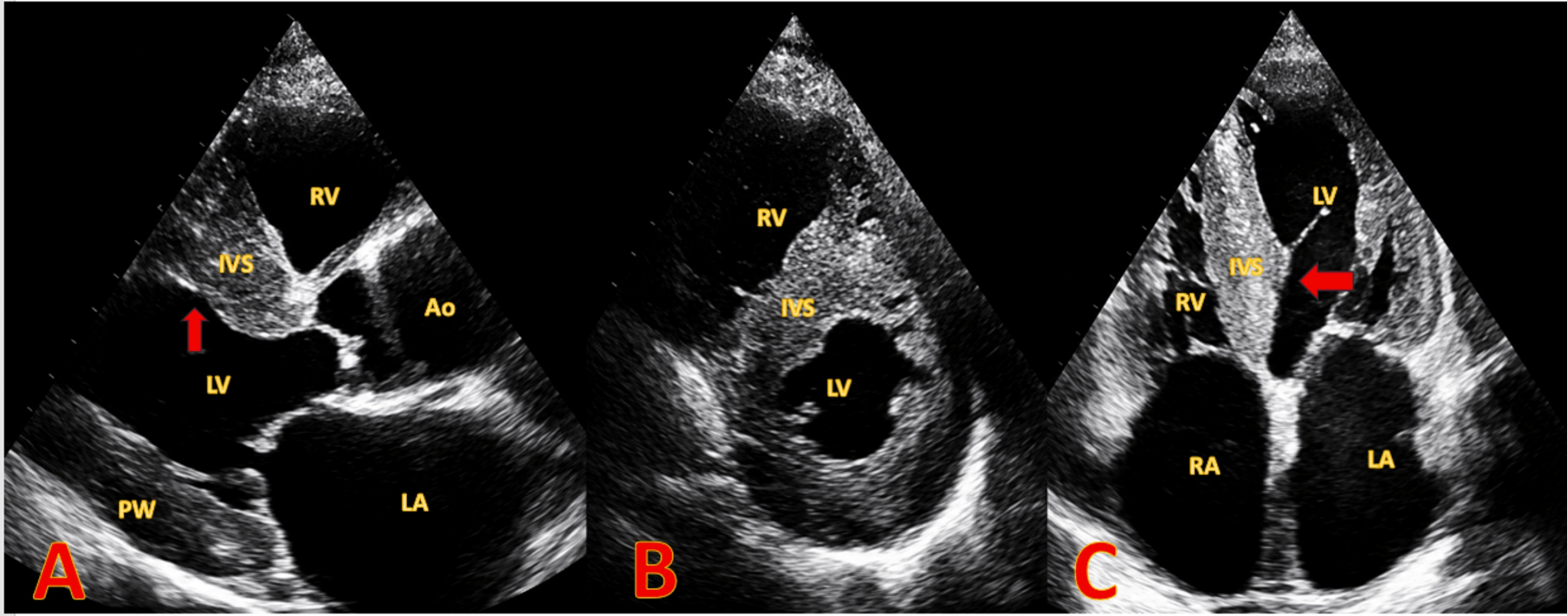
Transthoracic echocardiographic features suggestive of CA Transthoracic echocardiography demonstrating typical morphological features of CA. (A) Parasternal long-axis view showing marked left ventricular wall thickening and a granular sparkling appearance of the interventricular septum (red arrow). (B) Parasternal short-axis view demonstrating concentric left ventricular hypertrophy. (C) Apical four-chamber view showing biventricular hypertrophy with biatrial enlargement and marked thickening of the interventricular septum (red arrow). Ao: aorta, IVS: interventricular septum, LA: left atrium, LV: left ventricle, PW: posterior wall, RA: right atrium, RV: right ventricle, CA: cardiac amyloidosis

Bedside transthoracic echocardiography revealed marked concentric biventricular hypertrophy, with an interventricular septal thickness of 16 mm and a posterior wall thickness of 15 mm. Right ventricular free wall hypertrophy measuring 7 mm and biatrial enlargement were also present. The interventricular septum exhibited a characteristic sparkling or granular appearance (Figure 2A-2C).

**Figure 3:**
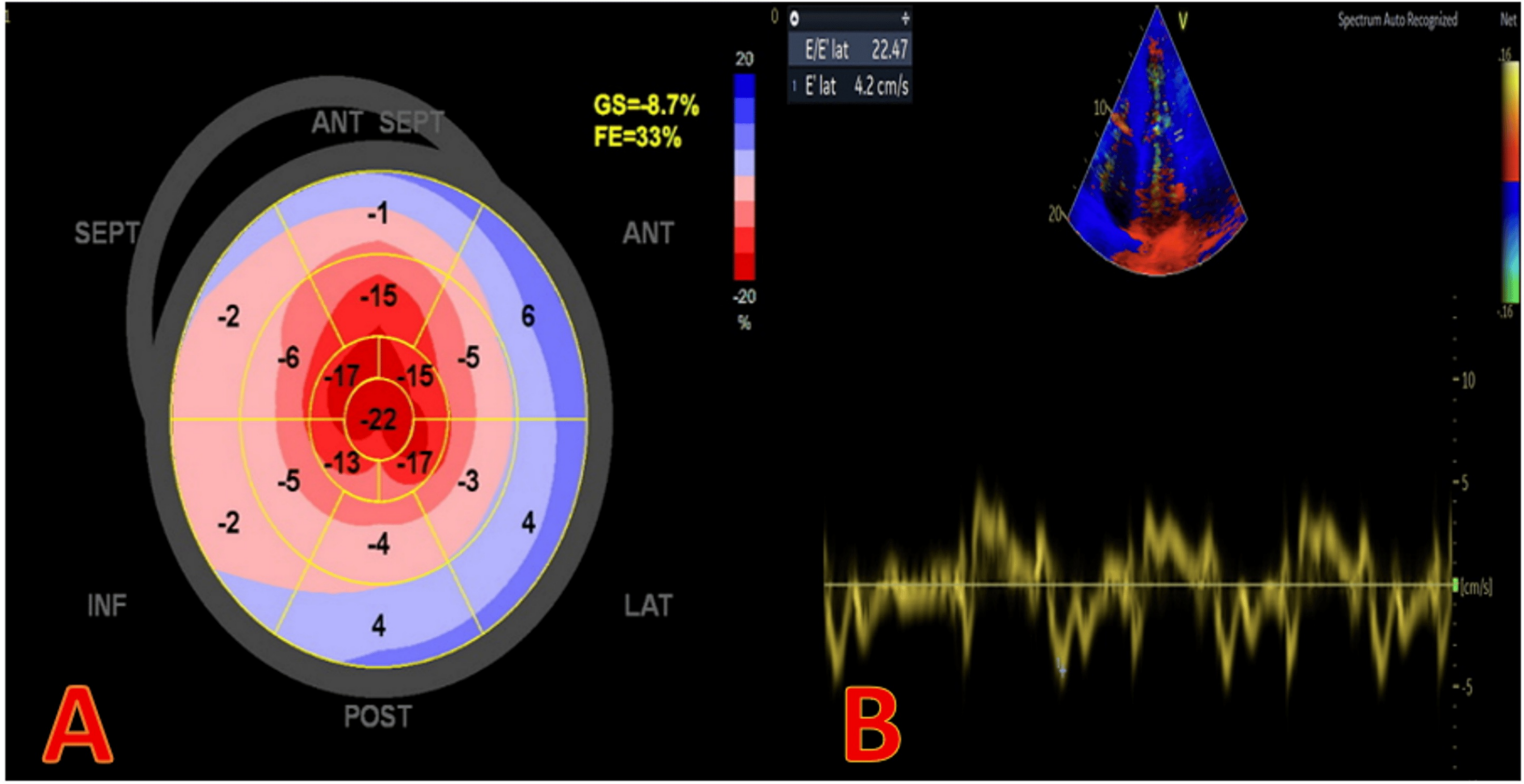
Functional echocardiographic findings suggestive of CA Functional echocardiographic assessment demonstrating characteristic findings of CA. (A) Left ventricular global longitudinal strain analysis showing severely impaired systolic function (GLS: −8.7%) with a typical apical sparing pattern on the bull's-eye map. (B) Tissue Doppler imaging demonstrating elevated left ventricular filling pressures with an E/e′ ratio of 22.47, consistent with restrictive left ventricular diastolic dysfunction. CA: cardiac amyloidosis

Doppler assessment demonstrated a restrictive left ventricular filling pattern with markedly elevated filling pressures (E/e′ ratio: 22.47) (Figure 3B). Left ventricular systolic function was impaired, with diffuse hypokinesia and a left ventricular ejection fraction of 33%. Speckle-tracking echocardiography showed severely reduced global longitudinal strain (-8.7%) with a characteristic apical sparing pattern on the bull's-eye map (Figure 3A).

The inferior vena cava was dilated (22 mm) and non-collapsible, suggesting elevated right atrial pressure. Moderate pulmonary hypertension was present, with a tricuspid regurgitation peak velocity of 3.0 m/s and an estimated pulmonary artery systolic pressure of 51 mmHg. No pericardial effusion was observed.

The combination of increased left ventricular wall thickness, biatrial enlargement, restrictive filling, markedly reduced global longitudinal strain with apical sparing, and complete atrioventricular block strongly suggested an infiltrative cardiomyopathy, particularly CA.

Laboratory investigations are summarized in Table 1. NT-proBNP was markedly elevated at 6,000 pg/mL, whereas high-sensitivity cardiac troponin remained within the normal range (6 ng/L). The complete blood count, renal function, and serum potassium were otherwise unremarkable. The markedly elevated NT-proBNP level indicated advanced myocardial involvement and was used to stage prognosis. The absence of troponin elevation suggested no acute myocardial injury and supported Mayo stage II classification.

**Table 1:** Laboratory investigations on admission Laboratory investigations performed on admission demonstrated markedly elevated NT-proBNP levels with preserved renal function, normal high-sensitivity cardiac troponin, and no significant hematological or electrolyte abnormalities. eGFR: estimated glomerular filtration rate, CKD-EPI: Chronic Kidney Disease Epidemiology Collaboration, NT-proBNP: N-terminal pro-B-type natriuretic peptide, hs-cTn: high-sensitivity cardiac troponin, WBC: white blood cell count

Parameter	Patient value	Reference range
White blood cell count	6,000/mm³	4,000-10,000/mm³
Hemoglobin	14 g/dL	12-16 g/dL
Platelet count	250,000/mm³	150,000-400,000/mm³
Blood urea	0.6 g/L	0.15-0.45 g/L
Serum creatinine	8 mg/L	5-11 mg/L
eGFR (CKD-EPI 2021)	79 mL/min/1.73 m²	>60 mL/min/1.73 m²
Serum potassium	4.0 mmol/L	3.5-5.0 mmol/L
NT-proBNP	6,000 pg/mL	<125 pg/mL
High-sensitivity cardiac troponin	6 ng/L	<14 ng/L

The patient was admitted to the cardiac intensive care unit, where she underwent emergency temporary transvenous pacing because of symptomatic complete atrioventricular block with profound bradycardia. The procedure resulted in hemodynamic stabilization and symptom resolution. After exclusion of reversible causes and persistence of complete atrioventricular block, a permanent dual-chamber pacemaker was implanted.

Given the strong suspicion of CA based on unexplained biventricular hypertrophy, biatrial enlargement, restrictive physiology, markedly reduced global longitudinal strain with apical sparing, and advanced conduction system disease, cardiac MRI (CMR) was subsequently performed after appropriate pacemaker programming to ensure MRI compatibility.

**Figure 4:**
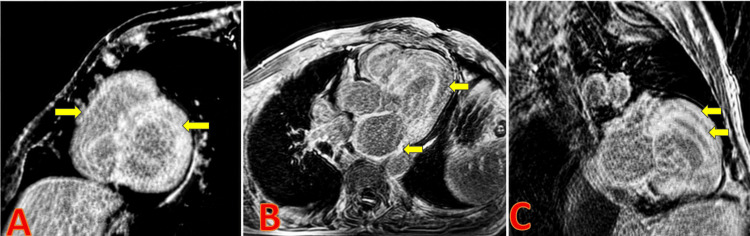
CMR imaging findings consistent with CA LGE CMR images demonstrating the characteristic diffuse subendocardial enhancement of ATTR-CM. (A) Short-axis view demonstrating diffuse circumferential subendocardial late gadolinium enhancement involving both the left and right ventricles (yellow arrows), consistent with biventricular amyloid infiltration. (B) Three-chamber (LVOT) view demonstrating diffuse subendocardial late gadolinium enhancement involving the left ventricle and left atrium (yellow arrows). (C) Long-axis view demonstrating diffuse circumferential subendocardial late gadolinium enhancement (yellow arrows), highly suggestive of CA. LGE: late gadolinium enhancement, LVOT: left ventricular outflow tract, CMR: cardiac magnetic resonance imaging, ATTR-CM: transthyretin cardiac amyloidosis, CA: cardiac amyloidosis

CMR demonstrated diffuse subendocardial late gadolinium enhancement, abnormal myocardial nulling, and extensive extracellular volume expansion, findings highly suggestive of CA (Figure 4).

Given the high clinical and imaging suspicion for CA, a hematological evaluation was performed to rule out AL amyloidosis. Serum protein electrophoresis showed no monoclonal spike, while serum and urine immunofixation electrophoresis and serum free light-chain (AL) measurements were all negative, making AL amyloidosis highly unlikely.

**Figure 5:**
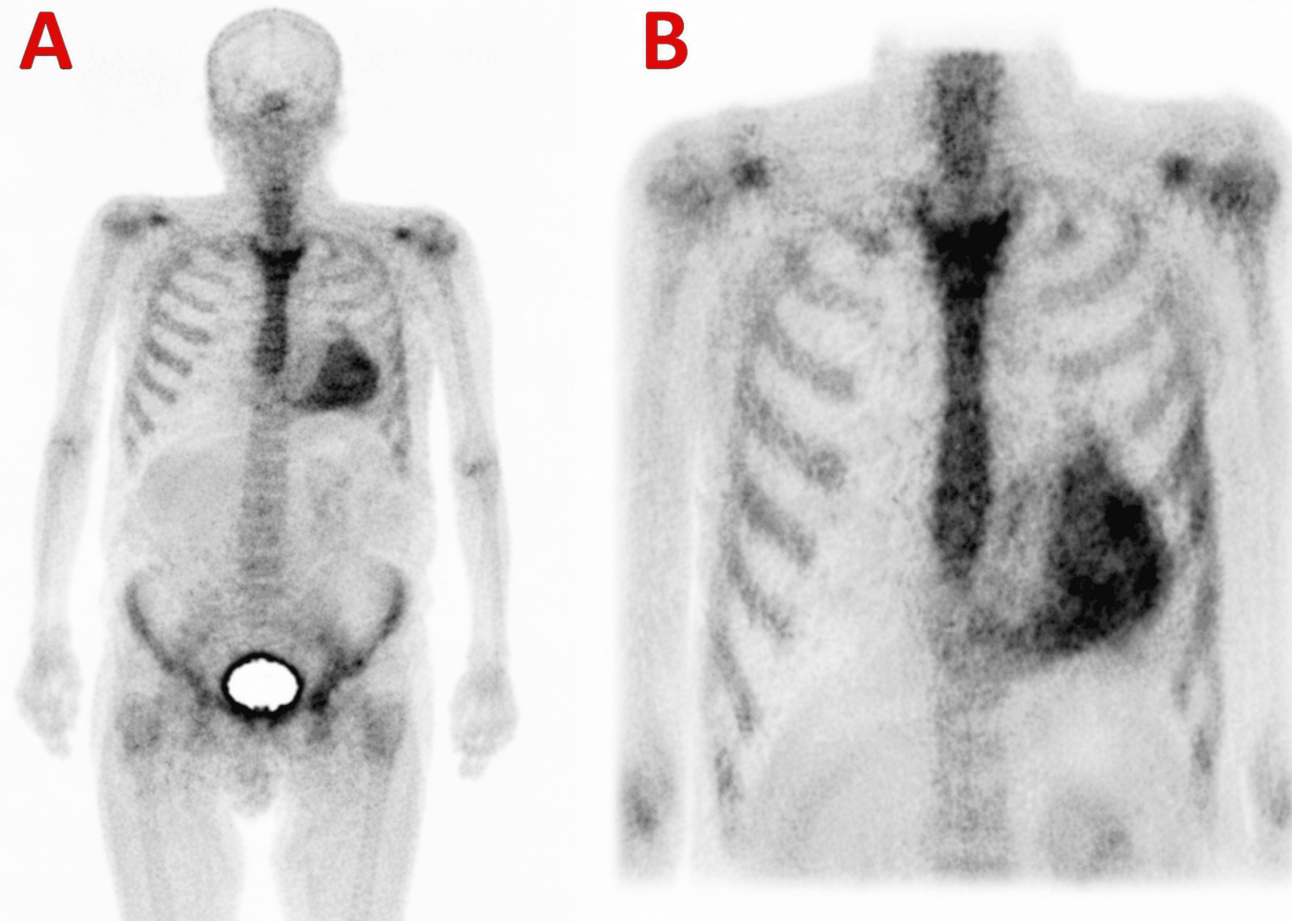
Bone scintigraphy confirming ATTR-CM 99mTc-HMDP bone scintigraphy demonstrating intense diffuse myocardial tracer uptake exceeding rib uptake, corresponding to a Perugini grade 3 pattern, is highly suggestive of ATTR-CM. (A) Whole-body anterior planar image showing prominent cardiac radiotracer uptake. (B) Magnified thoracic view demonstrating marked myocardial uptake with relative attenuation of rib uptake, confirming the diagnosis of ATTR-CM in the absence of a monoclonal gammopathy. ATTR-CM: transthyretin cardiac amyloidosis, 99mTc-HMDP: technetium-99m hydroxymethylene diphosphonate

Subsequently, bone scintigraphy with technetium-99m hydroxymethylene diphosphonate (99mTc-HMDP) demonstrated intense, diffuse myocardial tracer uptake that exceeded bone uptake, corresponding to a Perugini grade 3 pattern and highly suggestive of ATTR-CM (Figure 5). In the absence of evidence of a monoclonal gammopathy, these findings established the non-biopsy diagnosis of ATTR-CM.

According to the National Amyloidosis Center staging system, the patient was classified as stage II because of an NT-proBNP level >3000 pg/mL in the absence of significant renal dysfunction [6]. Similarly, according to the Mayo Clinic staging system, the patient was classified as stage II, as high-sensitivity cardiac troponin remained within the normal range despite elevated NT-proBNP levels [7].

The case was subsequently discussed during a multidisciplinary heart team meeting, and a decision was made to initiate tafamidis therapy. The indication for treatment was explained to the patient, who agreed to start therapy.

Genetic testing for TTR mutations was requested, as recommended in all patients with confirmed ATTR-CM, to distinguish hereditary ATTR from wild-type ATTR and to allow appropriate family screening if a pathogenic variant is identified.

At the three-month follow-up, the patient remained clinically stable, with no recurrence of syncope or heart failure symptoms. Blood pressure was 125/75 mmHg, permanent pacemaker interrogation confirmed normal device function, and tafamidis therapy was well tolerated.

This case emphasizes that complete heart block may be the first manifestation of ATTR-CM. It highlights the pivotal role of multimodality imaging in enabling rapid, accurate diagnosis, thereby facilitating the timely initiation of disease-modifying therapy.

## Discussion

CA is an infiltrative cardiomyopathy caused by extracellular deposition of misfolded amyloid fibrils within the myocardium, leading to progressive ventricular thickening, diastolic dysfunction with restrictive left ventricular filling, heart failure, arrhythmias, and conduction abnormalities [1]. ATTR-CM, once considered rare, is increasingly recognized owing to advances in multimodality imaging and the emergence of disease-modifying therapies [2].

Conduction abnormalities are common in CA and result from progressive amyloid infiltration of the sinoatrial node, atrioventricular node, and His-Purkinje system [3]. First-degree atrioventricular block, bundle branch block, and atrial arrhythmias are frequently encountered, whereas complete heart block as the inaugural manifestation of ATTR-CM remains distinctly uncommon [4]. In a large cohort of patients with ATTR-CM, approximately 9-10% eventually required permanent pacemaker implantation because of clinically significant conduction disease [5].

Only a limited number of case reports have described complete or high-grade atrioventricular block as the initial manifestation of CA (Table 2) [8,9]. Most reported patients presented with syncope or recurrent presyncope and ultimately required permanent pacemaker implantation before the diagnosis of amyloidosis was established. These observations suggest that unexplained advanced atrioventricular block in older adults should prompt careful evaluation for an underlying infiltrative cardiomyopathy, particularly in the presence of increased ventricular wall thickness.

**Table 2:** Previously reported cases of CA presenting with complete or high-grade atrioventricular block Summary of previously reported cases of CA presenting with complete or high-grade atrioventricular block, highlighting the clinical presentation, diagnostic approach, management, and outcomes. ATTR: transthyretin amyloidosis, ATTR-CM: transthyretin cardiac amyloidosis, CMR: cardiac magnetic resonance imaging, Tc-99m PYP: technetium-99m pyrophosphate, CA: cardiac amyloidosis

Author	Year	Age/sex	Initial presentation	Amyloidosis type	Diagnostic modalities	Management	Outcome
John et al. [8]	2019	51/F	Complete heart block and chronic pericardial effusion	CA (ATTR suspected)	Echocardiography, CMR, Tc-99m PYP scintigraphy	Permanent pacemaker	Clinical improvement
Rifai et al. [9]	2024	83/M	High-grade atrioventricular block and heart failure symptoms	ATTR-CM	Echocardiography, invasive hemodynamic evaluation, amyloidosis work-up	Permanent pacemaker and medical therapy	Clinical stabilization

Our patient shares several characteristics with previously reported cases, including advanced age, syncope at presentation, and the need for permanent pacing. However, unlike many earlier reports in which the diagnosis of amyloidosis was delayed, the combination of echocardiographic red flags, CMR findings, and bone scintigraphy enabled a rapid, noninvasive diagnosis of ATTR-CM. Early recognition enabled timely initiation of tafamidis therapy.

Echocardiography remains the cornerstone of the initial evaluation of patients with suspected CA [10]. The combination of unexplained left ventricular hypertrophy, biatrial enlargement, a restrictive left ventricular filling pattern, and impaired longitudinal systolic function should immediately raise suspicion for an infiltrative cardiomyopathy. Our patient exhibited marked biventricular hypertrophy, biatrial enlargement, elevated filling pressures, severely impaired global longitudinal strain, and a reduced left ventricular ejection fraction (33%), findings consistent with advanced myocardial involvement by ATTR-CM.

Speckle-tracking echocardiography further contributed to the diagnosis by demonstrating markedly reduced global longitudinal strain with relative preservation of apical strain, producing the characteristic “apical sparing” pattern. Phelan et al. first demonstrated the high diagnostic accuracy of this finding in differentiating CA from other causes of left ventricular hypertrophy [11]. Although not entirely specific, apical sparing is now considered one of the most valuable echocardiographic red flags for CA and has been incorporated into contemporary diagnostic algorithms [12].

The reduced left ventricular ejection fraction in our patient likely reflected advanced myocardial infiltration, as supported by diffuse biventricular late gadolinium enhancement, markedly impaired global longitudinal strain, and restrictive filling physiology. Reduced left ventricular ejection fraction has been associated with more advanced disease and poorer clinical outcomes in ATTR-CM, emphasizing the importance of early diagnosis and timely initiation of disease-modifying therapy before irreversible myocardial dysfunction develops.

CMR imaging has emerged as a major tool for tissue characterization in infiltrative cardiomyopathies. Diffuse subendocardial or transmural late gadolinium enhancement, abnormal myocardial nulling, and increased extracellular volume are highly suggestive of CA [13]. In our patient, CMR demonstrated diffuse biventricular subendocardial late gadolinium enhancement with abnormal myocardial nulling, findings that substantially reinforced the suspicion of amyloid infiltration.

The diagnosis was ultimately established noninvasively by bone scintigraphy with 99mTc-HMDP, which demonstrated intense myocardial tracer uptake consistent with a Perugini grade 3 pattern. Following the landmark study by Gillmore et al., grade 2 or 3 cardiac uptake on bone scintigraphy in the absence of a monoclonal gammopathy is sufficient to establish a non-biopsy diagnosis of ATTR-CM with excellent specificity [14]. Current European Society of Cardiology guidelines endorse this diagnostic approach and recommend systematic exclusion of AL amyloidosis before confirming ATTR-CM [15].

The differential diagnosis of increased left ventricular wall thickness in an elderly patient presenting with complete atrioventricular block includes hypertensive heart disease, sarcomeric hypertrophic cardiomyopathy (HCM), Fabry disease, and AL CA. Although the patient presented with elevated blood pressure (170/80 mmHg) on admission, she had no prior history of hypertension. Moreover, the degree of biventricular wall thickening, biatrial enlargement, restrictive left ventricular filling pattern, markedly reduced global longitudinal strain with relative apical sparing, and advanced atrioventricular conduction disease were disproportionate to that expected from hypertensive heart disease alone. Sarcomeric HCM was also considered; however, the absence of asymmetric hypertrophy, left ventricular outflow tract obstruction, or a family history of cardiomyopathy, together with the characteristic multimodality imaging findings, argued against this diagnosis. Fabry disease was considered less likely given the patient's age, the absence of characteristic extracardiac manifestations, and imaging findings not suggestive of Fabry cardiomyopathy. Finally, AL CA was excluded due to the absence of a monoclonal gammopathy, as demonstrated by negative serum and urine immunofixation electrophoresis and a normal serum free AL ratio. Taken together, the clinical presentation, multimodality imaging findings, and the absence of a monoclonal gammopathy strongly supported the diagnosis of ATTR-CM.

The pathophysiological basis of conduction disturbances in ATTR-CM lies in progressive amyloid infiltration and fibrosis of the conduction system, leading to impaired impulse generation and propagation [2]. Histopathological studies have demonstrated extensive amyloid deposition within the atrioventricular node and His-Purkinje system, explaining the high prevalence of conduction abnormalities and the frequent need for pacing therapy in these patients.

Although permanent pacemaker implantation effectively treats symptomatic bradyarrhythmias and prevents recurrent syncope, it does not modify the underlying infiltrative process [5]. The advent of disease-modifying therapies has profoundly changed the prognosis of ATTR-CM. The ATTR-ACT trial demonstrated that tafamidis significantly reduced all-cause mortality and cardiovascular hospitalizations while improving quality of life and functional capacity in patients with ATTR-CM [16]. In addition to transthyretin stabilizers, emerging transthyretin gene-silencing therapies, including patisiran, vutrisiran, and eplontersen, have demonstrated promising clinical benefits by suppressing hepatic transthyretin production and improving functional and clinical outcomes in patients with ATTR-CM [17]. Consequently, early diagnosis has become increasingly important because delayed recognition may deprive patients of potentially beneficial therapy.

This case highlights several important clinical messages. First, complete heart block may represent the inaugural manifestation of ATTR-CM, although this presentation remains rare. Second, unexplained advanced atrioventricular block in elderly patients, particularly when associated with increased ventricular wall thickness, should prompt consideration of an infiltrative cardiomyopathy. Finally, multimodality imaging combining echocardiography, CMR, and bone scintigraphy enables an accurate, rapid, and noninvasive diagnosis of ATTR-CM, thereby facilitating timely initiation of disease-modifying therapy.

To our knowledge, reports of ATTR-CM initially presenting with complete heart block remain uncommon. This case further emphasizes the importance of recognizing the association between unexplained conduction disease and infiltrative cardiomyopathy. It illustrates the pivotal role of multimodality imaging in achieving a rapid and accurate diagnosis, thereby enabling timely therapeutic intervention.

## Conclusions

This case illustrates that complete heart block, although uncommon, may represent the inaugural presentation of ATTR-CM. In elderly patients presenting with unexplained advanced atrioventricular conduction disease, particularly when accompanied by increased ventricular wall thickness or heart failure features, clinicians should maintain a high index of suspicion for an underlying infiltrative cardiomyopathy. This report further underscores the pivotal role of multimodality imaging, including echocardiography, CMR, and bone scintigraphy, in establishing a rapid, accurate, and noninvasive diagnosis of ATTR-CM. Prompt recognition is essential in the era of disease-modifying therapies, as timely diagnosis enables prompt initiation of targeted treatment and may improve patient outcomes. Increased awareness of this atypical presentation may facilitate earlier recognition of ATTR-CM in future patients and support timely initiation of disease-modifying therapy.
